# The contribution of epigenetics in Sjögren’s Syndrome

**DOI:** 10.3389/fgene.2014.00071

**Published:** 2014-04-03

**Authors:** Orsia D. Konsta, Yosra Thabet, Christelle Le Dantec, Wesley H. Brooks, Athanasios G. Tzioufas, Jacques-Olivier Pers, Yves Renaudineau

**Affiliations:** ^1^Research Unit EA2216 Immunology, Pathology and Immunotherapy, SFR ScinBios and Labex Igo “Immunotherapy Graft, Oncology”, Réseau Épigénétique du Cancéropole Grand Ouest, European University of Brittany BrestFrance; ^2^Department of Pathophysiology, School of Medicine, National University of AthensAthens, Greece; ^3^Department of Chemistry, University of South FloridaTampa, FL, USA; ^4^Laboratory of Immunology and Immunotherapy, Hôpital Morvan – Brest University Medical School BrestFrance

**Keywords:** Sjögren’s syndrome, DNA methylation, HERV, epithelial cells, microRNAs

## Abstract

Sjögren’s syndrome (SS) is a chronic autoimmune epithelitis that combines exocrine gland dysfunctions and lymphocytic infiltrations. While the pathogenesis of SS remains unclear, its etiology is multifunctional and includes a combination of genetic predispositions, environmental factors, and epigenetic factors. Recently, interest has grown in the involvement of epigenetics in autoimmune diseases. Epigenetics is defined as changes in gene expression, that are inheritable and that do not entail changes in the DNA sequence. In SS, several epigenetic mechanisms are defective including DNA demethylation that predominates in epithelial cells, an abnormal expression of microRNAs, and abnormal chromatin positioning-associated with autoantibody production. Last but not least, epigenetic modifications are reversible as observed in minor salivary glands from SS patients after B cell depletion using rituximab. Thus epigenetic findings in SS open new perspectives for therapeutic approaches as well as the possible identification of new biomarkers.

## INTRODUCTION

Sjögren’s syndrome (SS) is a chronic autoimmune disorder, affecting exocrine glands, mainly salivary and lacrimal glands, leading to the designation of SS as an autoimmune exocrinopathy or autoimmune epithelitis (Mavragani and Moutsopoulos, 2013). The clinical manifestations include dry mouth (xerostomia), dry eyes (keratoconjunctivitis sicca), and systemic features. Histological examination shows peri-epithelial mononuclear cell infiltrates in exocrine glands and parenchymal organs such as kidney, lung, and liver. Patients with SS have a 20–40 fold increased risk of developing lymphoma (Voulgarelis et al., 1999; Baimpa et al., 2009; Tobon et al., 2010). Furthermore, SS is characterized by the presence of circulating autoantibodies (Ab) against the sicca syndrome (SS)A/Ro and SSB/La ribonucleoprotein particles (Hu et al., 2011).

The pathogenesis of SS remains unclear. Its etiology is multifunctional and includes a combination of genetic predispositions, environmental factors, and epigenetic factors (Cobb et al., 2008; Mavragani and Moutsopoulos, 2010; Le Dantec et al., 2012; Liu and La Cava, 2013). Genetic factors associated with SS are particular HLA-DR allele subtypes and specific gene polymorphisms including STAT4, IL-12A, TNIP1, IRF5, BLK, and CXCR5 (Lessard et al., 2013). Most of these genetic mutations are far from DNA-coding regions and they are suspected to alter disease-susceptibility genes by altering their expression indirectly *via* an action on the epigenetic machinery (Dozmorov et al., 2014).

Epigenetics can be defined as changes in gene expression, that are inheritable and that do not entail changes in the DNA sequence. Epigenetics also explains how cells can differentiate into alternative cell types and how a phenotype can be passed from one cell to its daughter cells (Delgado-Vega et al., 2010). Epigenetic mechanisms are important to control the pattern of gene expression during development, the cell cycle, and in response to biological or environmental changes. As a consequence epigenetic dysregulations have been linked with autoimmune diseases including SS (Brooks et al., 2010; Renaudineau, 2010).

## EPIGENETICS AND CHROMATIN

### CHROMATIN

**FIGURE 1:**
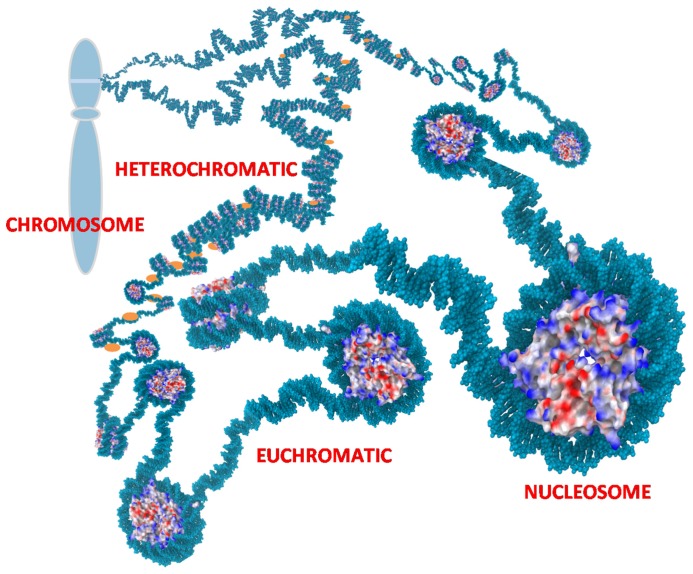
**Chromatin.** Nucleosomes are the repetitive unit in chromatin consisting of 145 bp of DNA wrapped around an octamer of histones. Charges on the histones are shown as blue (positive), red (negative), and white (neutral). The positive charges of histones facilitate binding to negatively charged DNA and thereby packaging the DNA to reduce the overall volume needed for the chromatin. Euchromatin is an extended form of chromatin that allows access to the underlying genes. Heterochromatin is a condensed form with histone H1 (orange) bound to the linker DNA between nucleosomes. This can compact the chromatin further and sequesters the underlying genes. The chromatin is further organized into loops that are usually tightly coiled and stacked into chromosomes but the loops can rapidly uncoil into extended states as depicted to allow access to sequestered genes.

Chromatin is located in the nucleus of eukaryotic cells, and consists of DNA, histones, transcription complexes, chromatin modifying enzymes, and other proteins to form chromosomes. Chromatin is divided into two forms, euchromatin and heterochromatin. Euchromatin is a loosely packaged form of chromatin that has a high concentration of genes and is often involved in active transcription or is at least potentiated for transcription. In contrast, heterochromatin is a denser packaging of DNA and proteins, which has many varieties. Heterochromatin is considered to be transcriptionally inactive since the genes are less accessible to transcription factors (**Figure 1**). Changes in chromatin’s structure are affected by chemical modifications of histones such as methylation and acetylation, and by the binding of other proteins and ions to the DNA. Most of the modifications are reversible, providing dynamics to the chromatin structure and activity that allows for opening of previously sequestered genes or suppression of previously active genes in response to stimuli and cell cycle progression.

### HISTONES

The basic unit of chromatin is the nucleosome, a nucleoprotein complex that consists of DNA wrapped around an octameric core of histones (two each of histones H2A, H2B, H3, and H4). Nucleosomes occur on average every 200 base pairs (bp) in chromatin with 145 bp (core DNA) in close contact with the histone core and another approximately 55 bp in the linker section (linker DNA) between nucleosome cores. The abundance of positively charged arginine and lysine residues in histones are sufficient to counter roughly half of the negative charges in the core DNA, thereby reducing the self-repulsion of the DNA and allowing a sevenfold compaction of the length held in DNA coils around the histone core. Histones are small globular proteins (11–15 kD) with flexible N-terminal tails that project from the nucleosome core. Epigenetic modification sites in the chromatin structure consist primarily of lysine, arginine and serine residues in the N-terminal tails of core histones that are post-translationally modified by acetylation, methylation, phosphorylation, ubiquitination/sumoylation, ADP ribosylation, deimination/citrullination, protein conjugation, or β-*N*-acetylglucosamination. As a consequence, the N-terminal tails can undergo modifications that add to or reduce the interactions of the histones on neighboring histones, DNA and nuclear proteins therefore affecting gene transcription (Huber et al., 2007; Dieker and Muller, 2010). For example, positively charged lysine residues can non-covalently bind to the negatively charged phosphate groups in the DNA but acetylation of a lysine residue removes the positive charge and reduces the histone-DNA interaction. An additional histone, histone H1, can bind to the linker DNA between nucleosomes allowing stacking of nucleosomes to provide further compaction of the chromatin. Histone H1 is more abundant in heterochromatin. The main histone modifications are listed in **Table 1**.

**Table 1:** Histone modifications as part of epigenetic control.

Modification	Targets	Enzyme involved	Effect	Reversible
Acetylation	Lysine	Histone acetyltransferases (HATs), Histone deacetylases (HDACs)	Acetylation removes positive charges and reduces histone-DNA interaction	Yes
Methylation	Arginine and lysine	Lysine methyltransferases, arginine methyltransferases, lysine demethylases	Methylation removes positive charges and reduces histone-DNA interaction	Yes
Phosphorylation	Serine, theonine, tyrosine	Kinases, phosphatases	Adds negative charges that can alter chromatin structure and accessibility	Yes
Ubiquitylation/sumoylation	Lysine	E1, E2, E3 add 8 kD ubiquitin or 12 kD SUMO, removal by isopetidase deubiquitin enzymes	Suppress gene expression, possible targeting of histone to proteosome for degradation	Yes
ADP Ribosylation	Glutamate and arginine	Poly-ADP-ribose-polymerase (PARP), poly-ADP-ribose-glycohydrolases	Mono-, poly-ribosylation restricts access, possible role in chromatin stabilization, such as in DNA repair	Yes
Deimination/citrrulination	Arginine, methylated arginine	Peptidyl arginine deiminase 4 (PAD4)	Removes positive charge of arginine, reduces histone-DNA interaction	No
Protein conjugation	Lysine	Transglutaminases	Covalent attachment of molecules to proteins and protein-protein crosslinking	No
β-N- acetylglucosamine	Serine, threonine	*O*-GlcNAc transferase, β-*N*-acetylglucosaminidase (*O*-GlcNase)	Sugar added by transferase, removed by *O*-GlcNcase	Yes

*Adapted from Bannister and Kouzarides (2011).*

Besides post-translational modifications of histones, epigenetic control can be affected by the histone variants present in chromatin. For example, macroH2A, a variant of the canonical H2A histone, greatly increases stability of nucleosomes, reduces chaperone-mediated exchange of H2A–H2B dimers and it suppresses acetylation of histones in nucleosomes (Bonisch and Hake, 2012). The inactive X chromosome in females is kept in a heterochromatic state due in part to a high incorporation of macroH2A. On the other hand, other histone variants provide epigenetic modification sites that allow nucleosome dynamics. The modifications can be as described above and can also include N-terminal proteolytic sites by which the histone is irreversibly altered to remove potential modifications and interactions of the N-terminal tail.

### DNA METHYLATION

DNA methylation is the covalent addition of a methyl group from the methyl donor *S*-adenosylmethionine (SAM) to the cytosine residues of CpG dinucleotides. It takes place at position 5 of the pyrimidine ring within the CpG dinucleotides. 70% of CpG sites in human DNA are methylated in pairs, while most of the unmethylated CpGs are situated in CpG islands. DNA methylation is catalyzed by DNA methyltransferases (DNMTs). DNMTs are enzymes, which transfer a methyl group to CpG sites in DNA molecules, thereby influencing transcriptional activity.

Three phylogenic DNMTs exist in mammals: DNMT1, DNMT3a, and DNMT3b. DNMT1 shows a preference for hemimethylated DNA *in vitro* are making it the main DNMT, whereas DNMT3a and DNMT3b methylate unmethylated and methylated DNA at an equal rate and serve in a *de novo* DNMT role.

The insertion of a methyl group into DNA leads to structural changes of chromatin and is associated with gene silencing, by binding methyl-CpG-binding proteins, such as MeCP2 and MBD2, which then recruit chromatin inactivation complexes containing histone deacetylases (HDACs) and histone methyltransferases (HMTs).

DNA methylation and histone modifications regulate gene expression by modulating the packaging of the DNA inside the nucleus. DNA methylation may also interfere with the binding of some transcription factors.

## INVOLVEMENT OF EPIGENETICS IN SS

### DEMETHYLATING AGENTS AND SS

A link between demethylating drugs and SS has been known since Cannat and Seligmann (1968) demonstrated that oral administration of hydralazine or isoniazid to mice for several weeks led to development of SS with immunological features of a systemic lupus erythematosus (SLE)-like disease including antinuclear antibody detection. This effect disappeared after discontinuation of the drug and variations were observed depending on the animal strain, age, and sex (Cannat and Seligmann, 1968). Because of their capacity to inhibit DNMT activity and to remove a methyl group from cytosines present in CpG islands, hydralazine and procainamide were referred to as demethylating agents (Taylor, 1968). Used to prevent hypertension, hydralazine was reported to induce SS in humans (Darwaza et al., 1988; Brooks, 2010).

Following this, Richardson’s group demonstrated that hypomethylated CD4^+^ T cells become autoreactive. Experiments with passive transfer of CD4^+^ T cells pretreated with either of two distinct DNMT inhibitors, 5-aza-2′-deoxycytidine or procainamide, into mice showed an induction of anti-dsDNA antibody (Ab) production with the characteristics of a severe immune complex glomerulonephritis (Quddus et al., 1993). Similar results were obtained when B cells were used instead of CD4^+^ T cells (Mazari et al., 2007).

### HERV AS SENSOR OF DNA DEMETHYLATION

#### Description

Human endogenous retroviruses (HERVs) have spread, by reversing the normal flow of genetic information from DNA to RNA, throughout the human genome and represent up to 7% of the genome (Balada et al., 2009). For the most part, HERV genes contain deletions, stop codons or frame shifts, such that no retroviral protein is expressed. However, a few copies have retained their ability to generate functional protein but are normally epigenetically suppressed.

The HERV-K and HERV-E families contain some of the most active retroviral elements in the human genome and increase the possibility that HERVs may have a role in human diseases (Renaudineau et al., 2005a, b). The HERV-K family is the only group of HERVs that can produce intact viral particles and it’s also one of the most transcriptionally active families as its members retain intact open reading frames (ORFs) which encode the viral particles (Tugnet et al., 2013).

Human endogenous retroviruses are believed to play a role in the pathophysiology of several autoimmune diseases, especially rheumatic diseases such as RA and SLE; and, in addition, numerous reports have identified HERV elements in salivary gland epithelial cells (SGECs) from SS patients (Le Dantec et al., 2012; Tugnet et al., 2013). As a consequence of this expression, retroviral antigens are produced, and antibodies (Ab) to Gag and Env regions of HERVs have been reported in patients with autoimmune diseases.

#### HERVs are overexpressed in salivary glands from SS patients

Garry et al. (1990) detected and identified human intracisternal A-type retroviral particle (hIAP), which is an endogenous antigen related to human immunodeficiency virus (HIV), in lymphoblastoid cells when co-cultured with homogenates of labial salivary glands from SS patients (Garry et al., 1990; Brookes et al., 1992; La Placa et al., 2004). At the same time, Brookes et al. (1992) mentioned that the human T cell lymphotropic virus (HTLV) related endogenous sequence, HRES1, was overexpressed in the epithelium of labial salivary glands obtained from patients with primary SS (pSS). HRES1 regulation by DNA methylation was recently provided (Garaud et al., 2009; Fali et al., 2013) Later, the HERV-K113, and HRV-5 retroviral elements were found overexpressed in pSS patients (Murovska et al., 2000; Moyes et al., 2005). More recently, using a RT-PCR approach we have observed that at least one HERV-E element was detected when testing labial salivary glands from SS patients (Le Dantec et al., 2012).

#### Anti-HERV autoantibodies of HERV elements in SS

Using immunoblotting to test HIV antigen, Talal et al. (1990) observed that 14/47 (30%) of pSS patients reacted with the HIV p24 capsid antigen although sera from these patients were not reactive against HIV gp41 and gp120 envelope antigens. Yamano et al. (1997) confirmed anti-HIV p24 reactivity in SS and provided, in addition, molecular and serological arguments to exclude HIV or HTLV1 viruses’ infections. The explanation came with Brookes et al. (1992) who used synthetic peptides derived from HRES1 endogenous retrovirus and demonstrated that anti-HRES1 Ab, detected in 35% of pSS patients, cross-react with the HTLV1 p19 capsid antigen. Similarly, Hishikawa et al. (1997) prepared a recombinant p30 gag protein from HERV-E clone 4-1 and related Western blotting experiments showed that anti-HERV 4-1 p30 gag Ab were detected in 35% of patients with pSS, whereas no HERV 4-1 anti-p30gag antibodies were found in healthy donors.

### X CHROMOSOME IN SS

There are many disagreements about the role of the X chromosome in the development of SS. First of all, the correlation between SS and the ratio of 9:1 between females and males strongly suggests involvement of the X chromosome and, second, there are numerous reports of trisomy X (47, XXX) and of a super female phenotype (mosaic of XXXXX/XXXX/XXX/XX/XO) in female patients with SS (Ozcelik, 2008).

Females inherit both maternal and paternal X chromosomes whereas men receive only the maternal X. The majority of X-linked genes are not sex-specific. Therefore, the majority of X-linked genes should have equivalent expression in males and females. X chromosome inactivation (XCI) is an epigenetic event initiated in each cell early in development that leads to the transcriptional silencing of one of the two X chromosomes in females. Consequently, the mechanism results in equal dosage of genes in males and females such that only one X chromosome is transcriptionally active in both sexes with equal amounts of the products of X-linked genes synthesized in cells (Brooks, 2010). The inactive chromosome is referred to as Xi and the active as Xa. The choice of which X chromosome to inactivate, the maternally derived or the paternally derived, is a random choice made in somatic cells early in the development of the embryo (Lessing et al., 2013).

As an example of the scale of chromatin, the eighth largest human chromosome, the X chromosome, has 150 million bp containing 1,098 genes, which is a relatively low gene density (Ross et al., 2005). Approximately 750,000 nucleosomes are involved in the packaging of the X chromosome. In the case of the inactive X chromosome, roughly 85% of the genes are kept silent in order to attain dosage compensation of X-linked genes. Besides the histone modifications described above, silencing of the inactive X chromosome exemplifies the use of two other major epigenetic mechanisms to suppress genes: DNA methylation and non-coding RNAs. DNA methylation in which a methyl group is added to cytosine bases in a DNA strand alters the topology in the major groove of DNA, which can mask binding sites from transcription factors, thereby suppressing expression of the underlying gene. RNA can also play a role in epigenetics in four different ways: (1) as a structural RNA, such as the X inactivation specific transcript (XIST RNA) which coats the inactive X chromosome; (2) as an agent recruiting histone modifying enzymes, such as deacetylases, to a site; (3) as a direct block to prevent transcription factors from initiating gene transcription; and (4) as an agent to hybridize with nascent RNA transcripts leading to degradation by ribonucleases that target double-stranded RNA (Bernstein and Allis, 2005).

It is known that women are affected more often by autoimmune diseases than men. In SLE, it was observed that increased DNA demethylation of the Xi was associated with over-expression of the X-linked CD40 ligand in CD4^+^ T cells purified from SLE women (Lu et al., 2007). Recently, Belkhir et al. (2013) demonstrated that membrane CD40L was overexpressed in *ex vivo* activated CD4^+^ T cells from female patients with pSS. However, this overexpression occurs through non-epigenetic regulatory mechanisms as demonstrated when using DNA demethylating drugs and when testing the DNA methylation status of the regulatory regions of CD40L. This discordance between SLE and SS patients, when testing CD4^+^ T cells, may be interpreted in terms of cellular specificity based on the observation that DNA demethylation affects predominantly SGEC (see below). In addition, it is important to note that a sexual dimorphism is not restricted to the immune cells but sexual dimorphism was also reported in epithelial cells from salivary glands (Konttinen et al., 2010).

### LYMPHOMA IN SS

Patients with pSS have a higher risk of developing lymphoma, and especially a non-Hodgkin B cell lymphoma. The pro-apoptotic death associated protein kinase (DAP-kinase) gene is regulated by DNA methylation and Toso et al. (2009) demonstrated that DNA demethylation of this gene was restricted to the SS subgroup, compared to the non-SS subgroup, in which the gene was aberrantly hypermethylated. Another argument linking DNA methylation and lymphoma predisposition in SS is related to the hypermethylation of the runt-related transcription factor (RUNX1) gene in CD4^+^ T cells from SS patients (Altorok et al., 2014).

Tobon et al. (2013) showed increased levels of Flt-3L in the sera of 18 patients with pSS and previous lymphoma in contrast with patients with pSS without lymphoma. Additionally, the analysis of biological parameters showed that lymphocytopenia, low levels of C4 and high levels of Flt-3L were associated with previous occurrence of lymphoma (Tobon et al., 2013). An epigenetic control of FLt3-L in SGECs is suspected and experiments should be conducted in SS patients to test this hypothesis.

## DNA DEMETHYLATION IN SS

### EPITHELIAL CELLS

Recently, we have reported in pSS patients, that global DNA methylation was reduced in labial salivary glands when comparing biopsy sections from pSS patients to controls, and that this defect was conserved when SGEC were primarily cultured (Thabet et al., 2013). At the molecular level, SGEC global DNA demethylation was associated with a decrease in the methylating enzyme DNMT1 and an increase in the demethylating partner Gadd45-alpha. These observations support an active DNA demethylation process in SGEC from SS patients. Furthermore, SGEC DNA methylation levels were inversely correlated with SS severity and B cell infiltration. The contribution of B cells in the demethylation process was further explored in SS patients treated with rituximab, a chimeric anti-CD20 monoclonal antibody from the TEARS study (Devauchelle-Pensec et al., 2007; Thabet et al., 2013). Indeed, global DNA methylation levels were higher 4 months after B cell depletion in comparison with the minor salivary gland biopsy obtained at the initiation of rituximab therapy, thus suggesting that DNA demethylation in SGEC may be attributed in part to the presence of infiltrating B cells. This hypothesis was confirmed *in vitro*, revealing in co-culture that B cell mediated DNA demethylation in SGEC works through an alteration of the Erk/DNMT1 pathway.

Treatment with 5-aza-2’-deoxycytidine results in the expression of the aquaporin 5 (AQP5) gene in the human salivary gland ductal cell line NS-SV-DC, and to the increased fluid secretion in the murine aging model C57BL/6CrSkc (Motegi et al., 2005; Yamamura et al., 2012). Accordingly, it was proposed to treat SS patients with DNA demethylating drugs in order to restore the abnormal salivary flux that characterized SS patients. However, AQP5 is not repressed but overexpressed in SGEC from SS patients, in agreement with the DNA demethylation status observed in these cells, and the defect is at the protein level with an abnormal subcellular localization as recently described (Lee et al., 2013).

Observing an increase of the bullous pemphigoid antigen 1 (BP320) protein coded by the epithelial splice variant of the dystonin (DST) gene in acinar cells from SS patients, Gonzalez et al. (2011) have explored DST promoter methylation status and observed a hypermethylation status. Further explorations are mandatory in order to reveal whether this dichotomy is related to an alternative promoter usage (Elliott et al., 2012) and/or post-translational modifications.

### T CELLS

A genome-wide analysis of DNA methylation in naïve CD4^+^ CD45RA^+^ T cells was recently performed in pSS patients. Among 485,000 CpG sites tested within the entire genome, 753 CpG motifs were differentially methylated with the majority (311 genes, 75%) being demethylated. Demethylated genes in pSS patients include lymphotoxin-α [previously known as tumor necrosis factor (TNF)-β] involved in T cell activation, genes implicated in the type I interferon pathway, and genes encoding for membrane water channel proteins.

Analysis of gene promoter DNA methylation status in CD4^+^ T cells has revealed a demethylation and overexpression of CD70 (TNSF7), and an absence of epigenetic regulation for interferon regulatory factor (IRF)5 (Yin et al., 2010; Gestermann et al., 2012).

### REGULATORY T CELLS

Lu’s group noted a DNA hypermethylation profile at the forkhead box P3 (FoxP3) promoter in pSS patients compared to healthy controls (Sharma et al., 2009). In turn, they observed significant decreases in expression of FoxP3 mRNA and protein in peripheral CD4^+^ T cells isolated from pSS patients but not in healthy controls. Such an observation is in agreement with the reports describing a quantitative and qualitative defective FoxP3 Treg subset in SS (Li et al., 2007; Alunno et al., 2013). Of note, FoxP3 Treg frequency in labial salivary glands correlates positively with the inflammation grade and risk factors for lymphoma development, such as C3 and/or C4 hypocomplementemia and cryoglobulinimia (Christodoulou et al., 2008; Sarigul et al., 2010).

## HISTONE MODIFICATIONS IN SS

In CD4^+^ T cells from SLE patients, it was demonstrated that DNA demethylation was associated with a global histone H3 and H4 hyperacetylation, and a histone H3 dimethylation at lysine 4 (Zhou et al., 2011). In additions, such modifications were linked with the development of specific autoantibodies as demonstrated with the lupus-derived monoclonal antibodies BT-164 and KM-2 that recognize histone H3 trimethylated lysine 27, and histone H4 acetylated lysine 8, 12 and 16, respectively (Dieker et al., 2007; van Bavel et al., 2011). Last but not least, HDAC inhibitors such as trichostatine A and suberoylanilide hydroxamic acid revert histone modifications and improve SLE disease without affecting autoantibody titers (Mishra et al., 2003; Reilly et al., 2004). These results suggest (i) that the DNA demetlylation observed in SGEC from SS patients is associated with histone modification, (ii) that among anti-histones autoantibodies detected in SS some of them target histone post-translational modifications, and (iii) that targeting histone modifications may be considered as a new treatment in SS.

## miRNA IN SS

### DESCRIPTION

miRNAs are small non-coding and single-strand RNA of 19–22 nucleotides in length, which regulate gene expression at the post-transcriptional level and are important in a wide range of physiological and pathological processes. miRNAs are generated in the nucleus as primary miRNA transcripts by RNA polymerase II, and are cleaved by an RNAse III enzyme, called Drosha. After, they are transported to the cytoplasm by exportin 5 for further processing by Dicer into mature miRNA duplexes;, they separated into single strands at the core of the multiprotein RNA-induced silencing complex (RISC) by argonaut proteins to generate miRNAs. Most miRNAs bind to the 3’ untranslated region (UTR) of the targeted mRNAs which leads to either mRNA degradation or translation or repression.

Dysregulation of miRNA expression is found to be associated with the onset and progression of inflammatory autoimmune diseases including SS. Consequently, in very recent years extensive research has been done on miRNAs and their connection with autoimmune diseases (Zare-Shahabadi et al., 2013).

### miRNA IN MINOR SALIVARY GLANDS FROM SS PATIENTS

miRNAs have been investigated in minor salivary glands from SS patients by different groups using different technical approaches (Alevizos et al., 2011; Kapsogeorgou et al., 2011; Tandon et al., 2012). From these studies it appears that miRNA expression is differentially expressed when SS patients were compared with controls. The predictive analysis of biologic pathways under miRNA control suggests regulation of the neurologic pathways controlling salivation (Alevizos et al., 2011), as well as the lack of transcriptional regulation of the two main SS’s autoantigens SSA/Ro and SSB/La by the miRNa let-7b which is repressed in pSS (Kapsogeorgou et al., 2011).

### miRNA IN PBMC AND EXOSOMES FROM SS PATIENTS

To date, only a few studies have been conducted on miRNAs in peripheral blood mononuclear cells (PBMCs) from SS patients and from SS-prone mouse models. According to Pauley’s group (Pauley et al., 2009), both miR146a and miR155 are up-regulated in response to the adaptive immune response in SS when testing humans and mice. Authors have shown, in pSS patients, that miR146a increases prior to disease onset in PBMCs, and during full-blown disease in the salivary glands thus suggesting that mir146a may be involved in early disease pathogenesis. In addition to being present in PBMC, miRNAs are also present in exosomes which are microvesicles secreted by a variety of cells including lymphocytes (Gallo et al., 2012).

Mir146a plays a critical role in increasing phagocytic activity and repressing inflammatory cytokine production in human monocytic THP1 cells. Mir146a is activated by NF kappa B, that controls the TLR/INF pathway through the TNF-associated factor 6 (TRAF6), the IL-1 receptor associated kinase (IRAK1), the signal transducer and activator of transcription 1 (STAT1), and IRF5 (Pauley et al., 2011). Additionally, Zilahi et al. (2012) measured the expression of miR146a and miR146b, and their target genes IRAK1, IRAK4, TRAF6 in PBMCs of patients with pSS and from healthy controls. By quantitative RT-PCR they found miR146a/b, and the gene of TRAF6, overexpressed in pSS patients, whereas the expression of IRAK1 was significantly decreased. They proposed that the TRAF6 gene contributes to the increased activation state of the NF-κB pathway by the involvement of PKCξ existing normally in the disease, and perhaps the TRAF6 gene could be a new biomarker of SS (Zilahi et al., 2012).

Experiments for mir155 have shown an effect on the response of toll-like receptors (TLRs) and interleukin-1 receptors (TIRs) that may affect the immune response. FoxP3 transcription factor, which is detected in a subset of T cells infiltrating SS salivary glands, has been shown to induce mir155 expression.

## CONCLUSION

The etiology and many aspects of the pathogenesis of SS are still unknown. Nowadays many researchers are trying to provide an explanation based on epigenetic studies as to how epigenetic modifications may influence the course of SS and affect autoreactivity (Thabet et al., 2012). Improvements in our knowledge of epigenetics give us the opportunity to find new causes that may explain the etiology of autoimmune diseases (Lu et al., 2010). Epigenetic studies offer us the opportunity to identify new targets with potential impact on the prevention or progression of autoimmune diseases.

## Conflict of Interest Statement

The authors declare that the research was conducted in the absence of any commercial or financial relationships that could be construed as a potential conflict of interest.
